# Hematobiochemical alterations and direct blood polymerase chain reaction detection of *Theileria annulata* in naturally infected crossbred cows

**DOI:** 10.14202/vetworld.2015.24-28

**Published:** 2015-01-06

**Authors:** Anita Ganguly, Vandna Bhanot, R. S. Bisla, Indrajit Ganguly, Harpreet Singh, S. S. Chaudhri

**Affiliations:** 1 Teaching Veterinary Clinical Complex, Lala Lajpat Rai University of Veterinary & Animal Sciences Regional Centre, Karnal, Haryana, India; 2 Disease Investigation Laboratory, Krishi Vigyan Kendra, Ambala, LUVAS, Haryana, India; 3 Division of Animal Genetics, National Bureau of Animal Genetic Resources, Karnal, Haryana, India

**Keywords:** biochemical parameter, hematological parameter, serum biochemistry, Tams1 gene, theileriosis

## Abstract

**Aim:**

The aim was to determine hemato-biochemical changes and rapid diagnosis of *Theileria annulata* in naturally infected crossbred cows.

**Materials and Methods:**

Blood samples from lactating crossbred cows (n=40) between 3 and 7 years of age and showing clinical signs of tropical theileriosis were collected, with or without anticoagulant, and analyzed for tropical theileriosis by direct smear, direct blood polymerase chain reaction (PCR) detection of merozoite-piroplasm surface antigen (Tams1) gene specific amplicon, estimation of hematological and biochemical parameters. Healthy crossbred cows (n=6), examined free from hemoprotozoan infections were included as control.

**Results:**

The infected crossbred cows revealed significantly (p<0.001) lower values of total erythrocytic counts (4.46±0.2 × 10^6^/µL), hemoglobin (Hb 6.025±0.39 g%), packed cell volume (17.05±1.1%), mean corpuscular volume (37.94±1.70 fL) and mean corpuscular Hb (13.5±0.48 pg; p<0.002) compared with healthy control. The serum samples of infected cows revealed profound (p<0.05) hyponatremia (Na 133.21±2.36 mEq/l) and hypocalcemia (Ca 8.39±0.34 mg%). Infected crossbred cows showed a significant increase (p<0.05) of mean serum activity of alanine aminotransferase (61.45±13.36 U/L), aspartate aminotransferase (146.1±20.97 U/L), blood urea nitrogen (28.26±3.90 mg%), creatinine (1.55±0.13 mg%), direct bilirubin (0.33±0.04 mg%; p<0.001) and lactate dehydrogenase (3001.32±167.0 U/L; p<001). Blood direct PCR revealed a 721-bp fragment amplified from the target gene encoding 30-kDa major merozoite surface antigen of *T. annulata* using specific primer pairs. This assay was positive for all the infected animals.

**Conclusion:**

The assessments of hemato-biochemical parameters in *T. annulata* infected crossbred cows may be useful in understanding disease pathogenesis, prognosis and corrective measures for supportive therapy. Moreover, blood direct PCR can reliably be used for rapid detection of *T. annulata* in conjunction with microscopic examination.

## Introduction

Tropical theileriosis, a tick-borne hemoprotozoan disease caused by *Theileria annulata* and transmitted by *Hyalomma* spp., is one of the most devastating blood parasites affecting crossbred cattle. It is characterized by lymphadenopathy, splenomegaly, fever, anemia, weakness and loss of body weight [1,2]. About 250 million cattle in many countries, including Iran, Turkey, India, and China are at a risk of this disease, which is incurring heavy economic losses to the livestock owners through mortality and loss in productivity [3]. Much of the pathology in theileriosis is due to intra-lymphocytic schizogony [4] and associated alteration in biochemical and hematological parameters [3,5].

Diagnosis of clinical *T. annulata* infection in bovines is usually based on the detection of macroschizonts in lymphocytes and piroplasms in red blood cells in stained lymph node biopsy and blood smears, respectively. Serological tests such as indirect immunoflurescent antibody test (IFAT) have also been used to detect circulating antibodies against antigens of piroplasms and/or macroschizonts [6]. The cross-reactivity with antibodies directed against other *Theileria* species limits the specificity of IFAT [7]. Moreover, antibodies tend to disappear in long-term carriers although *Theileria* piroplasms persist. Hence, animals with negative serological test but positive for *T. annulata* piroplasms can pose a major threat for crossbred cattle. Molecular diagnostic assay with polymerase chain reaction (PCR) has allowed the development of sensitive diagnostic assay for *T. annulata* [8].

The present study was aimed at determination of hematobiochemical alterations and direct blood PCR detection of *T. annulata* in naturally infected crossbred cows.

## Materials and Methods

### Ethical approval

Research review committee of Lala Lajpat Rai University of Veterinary and Animal Sciences (LUVAS), Haryana, the primary author’s institution, approved the present study.

### Sample collection

**Figure-1:**
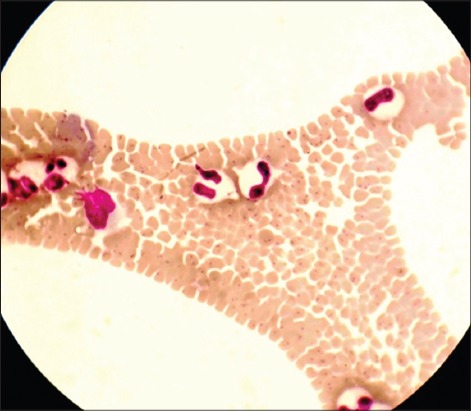
Microscopic examination of giemsa stained blood film showing *T. annulata* piroplasms in the erythrocytes.

Lactating crossbred cows (3-7 years) brought to out-patient department of LUVAS Regional Centre at Uchani, Karnal during the period of July, 2012-June, 2013 and showing clinical signs (Fever, anemia, swollen lymph nodes, loss in body weight etc.) similar to tropical theileriosis were included in the present study. Crossbred cows (n=40) showing ≥5% parasitemia constituted the infected group; whereas, six healthy crossbred cows found free from hemoprotozoan infections by microscopic examination, and direct blood PCR assay were included in healthy control group. The blood samples from infected and healthy control groups were collected in vials with or without anticoagulant (ethylenediaminetetraacetic acid). Blood smears were prepared immediately after collection from the anticoagulated blood, stained with Giemsa stain and examined microscopically for the presence of *T. annulata* (Figure-1). Blood collected in anticoagulant vials was used for hematological examination and PCR assay. The coagulated blood samples were centrifuged at 5000 rpm for 15 min and the supernatant (serum) was collected for biochemical estimations.

### Estimation of hematological parameters

Approximately, 1.5 ml of blood sample collected with anticoagulant was analyzed for hematological parameters including haemoglobin (Hb g/dL), packed cell volume (PCV %), total erythrocyte count (TEC × 10^6^/µL), total leukocyte count (TLC × 10^3^/µL) and differential leukocyte count as per method described [9]. Mean corpuscular volume (MCV), mean corpuscular hemoglobin (MCH) and mean corpuscular hemoglobin concentration (MCHC) were calculated [10].

### Biochemical assays

Total serum protein (TSP), glucose (Gl), calcium (Ca), phosphorus (P), alanine aminotransferase (ALT), aspartate aminotransferase (AST), gamma-glutamyl transpeptidase, lactate dehydrogenase (LDH), blood urea nitrogen (BUN), creatinine (Cr), total bilirubin (TBIL) and direct bilirubin (DBIL) were measured by semi auto analyzer (3000 Evolution, Biochemical Systems International, Italy) using commercial kits (Siemens Healthcare Diagnostics Ltd. Baroda, India). Sodium (Na) and potassium (K) levels of serum samples were determined by a micro-controller based Flame Photometer-128 (Systronics, India).

### Blood direct PCR

Blood direct PCR was performed by taking 2 μl of whole blood sample and targeting a gene encoding 30-kDa merozoite-piroplasm surface antigen Tams1 (Tams1) gene of *T. annulata* using specific primer pairs (forward 5’-GTAACCTTTAAAAACGT-3’; Reverse 5’-GTTACGAACATGGGTTT-3’) as previously described [8]. Initially, PCR assay was standardized by using positive blood samples with ≥80% parasitemia as per instruction of phusion blood direct PCR kit (Thermo Fisher Scientific, India, Pvt. Ltd.). For the validation of PCR assay and its detection sensitivity, the same direct blood PCR was carried out on blood samples of 20 *T. annulata* infected animals having varying degree of parasitemia.

Briefly, PCR was carried out in a thermal cycler (Veriti™, Applied Biosystem) with a final reaction volume of 20 µl containing 2 μl of whole blood, 1 × Phusion blood PCR buffer, 0.5 μM of each primer and 0.4 μl phusion blood II DNA polymerase. A negative control without template (whole blood) was always included to rule out PCR carryover. The PCR conditions were initial denaturation at 98°C for 5 min; followed by 35 cycles of 98°C for 2 s, 45°C for 30 s and 72°C for 30 s; with a final extension step of 72°C for 1 min. After completion of PCR, tubes were centrifuged at 1000 ×*g* for 2 min to collect the clear supernatant. PCR products (supernatant) were analyzed by electrophoresis on 1.5% agarose gel, containing ethidium bromide, and documented under gel documentation system (Gel Doc XR+, Bio-Rad) to confirm the fragment sizes.

### Statistical analysis

The differences of means of serum values between *T. annulata* infected and healthy control groups were compared using Student’s t-test [11]. Statements of statistical significance were based on 1 and 5% level of significance.

## Results and Discussion

The hematological values of *T. annulata* infected and healthy control crossbred cows have been presented in Table-1. The infected group showed significantly (p<0.001) lowered values of TEC (4.46±0.2), Hb (6.025 ±0.39), PCV (17.05 ±1.1) MCV (37.94 ±1.70) and MCH (13.5±0.48; p<0.002) than healthy control animals indicating normocytic hypochromic anemia. Similar findings have already been reported [12,13] and normocytic hypochromic anemia has found to be associated with theileriosis, while normocytic normochromic anemia with babesiosis [14]. The main reason may probably because of severe damage caused by the organisms inside the red blood cells during their multiplication. The cows of infected group also showed a significant increase in monocytes (p<0.001), eosinophilic counts (p<0.008) as well as a decrease (p<0.05) in neutrophilic counts (Table-1). A slight non-significant (p>0.05) increase of TLC and lymphocytic counts was observed in infected crossbred animals. The changes in leukogram might be attributed to persistent harmful effects of toxic metabolites of *Theileria* on the hemopoietic organs especially bone marrow and their interference with the process of leukogenesis. Relative increase in the number of lymphocytes and monocytes reflects compensatory mechanism as target cells in response to their invasion with *Theileria* protozoan [15]. The lymphocytosis may be due to intra-lymphocytic theilerial parasites transforming the host cells, leading to clonal growth of lymphocytes [16].

**Table-1:** Hematological parameters of normal and *T. annulata* infected crossbred cattle.

Parameters	Infected cows (*n*=40)		Healthy control (*n*=6)		Reference range ¥	p value
	
	Mean±SEM	Range	Mean±SEM	Range		
Hb (g/dL)	6.025±0.39	2.0-10.0	10.566±0.41	9.5-12.4	8.0-15.0	0.000**
PCV (%)	17.05±1.1	6.0-29.0	30.5±0.34	30.0-32.0	24.0-46.0	0.000**
TEC (×10^6^/µL)	4.46±0.20	2.11-7.10	7.28±0.16	6.72-7.89	5.0-10.0	0.000**
MCV (fL)	37.94±1.70	20.97-48.09	41.94±0.90	39.29-44.64	40.0-60.0	0.001**
MCH (pg)	13.5±0.48	9.48-16.03	14.48±0.26	13.96-15.71	11.017.0	0.002*
MCHC (%)	36.71±0.84	32.14-43.75	34.61±1.06	31.67-40.0	30.0-36.0	0.194
TLC (×10^3^/µL)	6.99±0.32	4.4-12.9	6.03±0.51	4.60-7.60	4.0-12.0	0.147
Lymphocyte (%)	60.5±2.39	24.0-85.0	51.66±3.70	40.0-60.0	45.0-75.0	0.073
Monocyte (%)	1.65±0.12	0.00-3.0	1.00±0	0.0-2.0	2.0-7.0	0.000**
Neutrophil (%)	35.85±2.31	13.0-70	46.5±3.99	37.0-60.0	15.0-45.0	0.047*
Eosinophil (%)	2.0±0.16	0.00-4.0	1.0±0.26	0.0-2.0	0-20.0	0.008*

* Infected and uninfected cattle significantly different at p<0.05.

** Significant at p<0.001.

¥: Reference range adopted from [23]. Hb=Hemoglobin, PCV=Packed cell volume, TEC=Total erythrocyte count, MCV=Mean corpuscular volume, MCH=Mean corpuscular haemoglobin, MCHC=Mean corpuscular hemoglobin concentration, TLC=Total leukocyte count, SEM=Standard error of mean, *T. annulata: Theileria annulata*

Serum samples of *T. annulata* infected cows showed significantly lower (p<0.05) values of Na (133.21±2.36) and Ca (8.39±0.34) than that of healthy control; whereas, K level (4.07±0.13) was significantly (p<0.05) higher than that of healthy group (Table-2). A significant (p<0.05) increase in the activity of ALT (61.45±13.36), AST (146.1±20.97), BUN (28.26± 3.90) and Cr (1.55±0.13), was observed in cows of infected group in comparison to healthy control (Table-2). In addition, we also observed significant (p<0.001) increase of DBIL (0.33±0.04) and LDH (3001.32±167.0) levels in the infected group (Table-2). In the present study, serum sodium levels decreased in all the infected animals compared with control group that is in accordance with recent research finding [17]. Animals suffering from theileriosis generally display increase in activity of ALT, AST, TBIL, BUN and icterus index, with a decrease in TSP [18]. Hypocalcaemia and hyponatremia in *Theileria* infected cows are probably due to decreased dietary intake, intestinal malfunction, and kidney damage whereas increased serum activities of AST and ALT are closely associated with hepatic injury caused by the protozoa [19]. Furthermore, a significant increase in the serum AST and ALT activities may also be due to muscle trauma caused by prolonged clinical recumbency in theileriosis [20]. In the present study, we observed a significant (p<0.001) increase of LDH activity in the infected group. This may probably be due to tissue damage of liver and kidneys resulting from infected lymphoid cells [19]. Hematological and sero-biochemical alterations are the indicators of severity of disease and are considered to be good tools for the diagnosis, prognosis for effective therapy [21,22].

**Table-2:** Hematological parameters of normal and *T. annulata* infected crossbred cattle.

Parameters	Infected cows (n=40)		Healthy control (n=6)		p value
	
	Mean±SEM	Range	Mean±SEM	Range	
Ca (mg/dL)	8.39±0.34	2.6-12.0	9.21±0.11	9.0-9.7	0.026*
P (mg/dL)	4.98±0.22	2.7-8.9	4.51±0.21	4.0-5.4	0.137
Na (mEq/L)	133.21±2.36	84.34-156.4	143.05±2.30	138.06-152.9	0.007*
K (mEq/L)	4.07±0.13	2.52-5.57	3.47±0.36	3.06-4.03	0.009*
ALT (U/L)	61.45±13.36	15-506	27.66±3.52	18.0-42.0	0.018*
AST (U/L)	146.1±20.97	23-564	84.66±1.36	80.0-89.0	0.006*
GGT (U/L)	38.17±5.01	10-141	33.16±3.25	20.0-41.0	0.408
Glucose (g/dL)	54.79±2.95	16.7-89.6	48.71±2.59	41.3-59.0	0.136
TSP (g/dL)	7.19±0.27	1.6-10.9	7.12±0.29	6.0-7.8	0.849
BUN (mg/dL)	28.26±3.90	8.5-146.9	17.38±1.18	12.9-21.5	0.009*
Cr (mg/dL)	1.55±0.13	0.4-5.4	0.73±0.19	0.4-1.7	0.006*
TBIL (mg/dL)	0.77±0.09	0.1-2.8	0.67±0.18	0.2-1.2	0.609
DBIL (mg/dL)	0.33±0.04	0.0-0.9	0.16±0.02	0.1-0.2	0.000**
LDH (U/L)	3001.32±167.0	1800-6640	1111.33±52.30	900-1214	0.000**

* Infected and uninfected cattle significantly different at p<0.05.

** Significant at p<0.001. Ca=Calcium, P=Phosphorus, Na=Sodium, K=Potassium, TSP=Total serum protein, ALT=Alanine aminotransferase, AST=Aspartate aminotransferase, GGT=Gammaglutamyltranspeptidase, BUN=Blood urea nitrogen, Cr=Creatinine, TBIL=Total bilirubin, DBIL=Direct bilirubin, LDH=Lactate dehydrogenase, SEM=Standard error of mean, *T. annulata: Theileria annulata*

**Figure-2:**
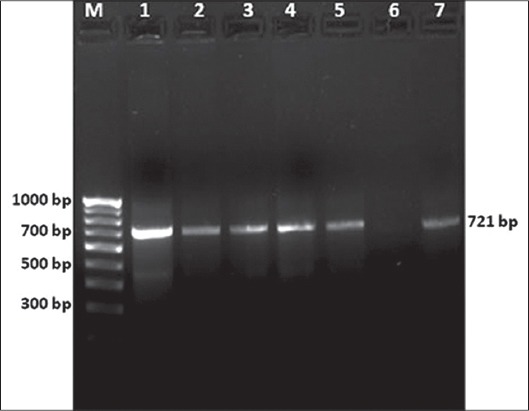
Detection of 721 bp amplified DNA from *T. annulata* infected blood samples using direct blood polymerase chain reaction method and resolved in a 1.5% agarose gel. M: 100 bp DNA ladder; Lane 1: Positive control (where ≥80% red blood cells were infected); Lane 2-5 and 7: samples with various degree of parasitaemia; Lane 6: Negative control.

The direct blood PCR assay in the present investigations produced one 721-bp fragment, pertaining to merozoite-piroplasm surface antigen Tams1 (Tams1) gene of *T. annulata* and clearly visible on gel (Figure-2). The fragment was further confirmed by sequencing. The animals confirmed positive by stained blood smears were found to be positive with direct blood PCR assay, thus confirming its utility in rapid diagnosis for detection of *T. annulata* with high specificity and sensitivity.

## Conclusion

From the findings of present study, it can be concluded that the observed changes in hematological and biochemical values in *T. annulata* infected crossbred cows are useful in understanding disease pathogenesis, prognosis and corrective measures for supportive therapy. The blood direct PCR can reliably be used for rapid detection of *T. annulata* in conjunction with microscopic examination.

## Authors’ Contributions

AG, SSC and IG designed the study. AG and VB conducted the laboratory analysis. AG and SSC analyzed the data. RSB and HS clinically diagnosed the animal for theileriosis. AG and IG drafted the manuscript. All authors read and approved the final manuscript.
